# Is HDL cholesterol protective in patients with type 2 diabetes? A retrospective population-based cohort study

**DOI:** 10.1186/s12967-020-02357-1

**Published:** 2020-05-06

**Authors:** Giovanni Fanni, Rosalba Rosato, Luigi Gentile, Matteo Anselmino, Simone Frea, Valentina Ponzo, Marianna Pellegrini, Fabio Broglio, Francesca Pivari, Gaetano Maria De Ferrari, Ezio Ghigo, Simona Bo

**Affiliations:** 1 Department of Medical Sciences, University of Turin, Corso AM Dogliotti, 14 10126 Turin, To Italy; 2 Department of Psychology, University of Turin, Turin, Italy; 3 Diabetic Clinic, Hospital of Asti, Asti, Italy; 4 Cardiology Unit, Città della Salute e della Scienza Hospital and University of Turin, Turin, Italy; 5 Department of Health Sciences, University of Milan, Milan, Italy

**Keywords:** High-density lipoprotein, Type 2 diabetes mellitus, Infectious diseases, Mortality

## Abstract

**Background:**

The protective role of high HDL cholesterol levels against cardiovascular diseases has been recently questioned. Limited data are available on this specific topic in patients with type 2 diabetes mellitus (T2DM). We aimed to evaluate the association of HDL cholesterol concentrations with all-cause and cause-specific mortality in a historical cohort of T2DM patients with 14 years of follow-up.

**Methods:**

This is a retrospective population-based cohort study involving 2113 T2DM patients attending the Diabetic Clinic of Asti. Survival analyses were performed to assess hazard ratios for overall and specific-cause mortality by HDL cholesterol tertiles, using the middle HDL cholesterol tertile as a reference.

**Results:**

The mean age was 66 ± 11 years; 51.4% of patients had low HDL-cholesterol levels. After a 14-year follow-up, 973/2112 patients had died (46.1%). The HDL cholesterol tertile cut-off points were 37.5 and 47.5 mg/dL (males) and 41.5 and 52.0 mg/dL (females). No associations between lower and upper HDL cholesterol tertiles respectively and all-cause (HR = 1.12; 95% CI 0.96–1.32; HR = 1.11; 0.95–1.30), cardiovascular (HR = 0.97; 0.77–1.23; HR = 0.94; 0.75–1.18) or cancer (HR = 0.92; 0.67–1.25; HR = 0.89; 0.66–1.21) mortality were found. A significantly increased risk for infectious disease death was found both in the lower (HR = 2.62; 1.44–4.74) and the upper HDL-cholesterol tertiles (HR = 2.05; 1.09–3.85) when compared to the reference. Individuals in the upper tertile showed an increased risk for mortality due to diabetes-related causes (HR = 1.87; 1.10–3.15).

**Conclusions:**

Our results corroborate the hypothesis that HDL cholesterol levels are nonprotective in T2DM patients. The U-shaped association between HDL-cholesterol levels and mortality associated with infectious diseases should be verified by further studies.

## Background

The level of HDL cholesterol is influenced by common and rare genetic variants, elevated blood triglyceride levels, obesity, smoking, sedentarism and alcohol consumption [1]. Recent findings have shown that HDL function can be negatively affected by aging, systemic diseases and/or inflammatory statuses without changes in HDL cholesterol concentrations [2–4]. This complicates the interpretation of the observed associations between HDL cholesterol levels and cardiovascular (CV) risk [5].

Evidence from epidemiological studies has raised doubt about the protective role of high HDL cholesterol levels against CV disease (CVD) risk; intriguingly, U-shaped associations between HDL cholesterol concentrations and CVD risk and mortality have been reported [6–11]. The conformational and functional properties of HDL particles may be altered in individuals with extremely high HDL cholesterol and HDL functionality may be compromised such that those particles no longer function but rather cause harm [1].

Patients with type 2 diabetes mellitus (T2DM) are characterized by an atherogenic lipidic profile with reduced HDL cholesterol concentrations, even though the precise underlying mechanisms have not been completely understood [12, 13]. HDL particles are known to be highly heterogeneous in structure and content. The chronic inflammatory state and non-enzymatic apolipoprotein glycation that occur in T2DM contribute to altering the relative composition of HDL and impairing HDL-associated anti-inflammatory and anti-atherogenic properties [2, 14–17], thus hampering the protective effect of HDL on vessels.

To the best of our knowledge, only one study has been carried out to analyze the associations among HDL cholesterol, CV risk and mortality in patients affected by T2DM, showing increased all-cause and CV mortality in patients in the two extreme HDL cholesterol quartiles in the presence of LDL cholesterol < 77 mg/dL. This association was not found in patients with higher LDL cholesterol levels [8].

Despite the well-known lower HDL cholesterol levels in fasting or postprandial states that characterize about approximately half of all individuals with T2DM [18, 19], there are no specific HDL cholesterol cut-off values for these patients. Furthermore, no specific goals for HDL cholesterol levels have been defined, even in the most updated guidelines for the general population [20]. Finally, the heterogeneity of cut-off values and reference range limits among studies on this topic impaired adequate comparison and interpretation of existing literature.

The aims of this retrospective population-based study were to evaluate the association of HDL cholesterol concentrations with overall and cause-specific mortality in a historical cohort of T2DM patients after a 14-year follow-up in a real-world setting. The advantages of this cohort were the low number of patients treated with statins/fibrates and the unavailability of the new hypoglycemic drugs that greatly impact studied outcomes, thus reducing the likelihood of those confounding factors affecting the results.

## Materials and Methods

### Study design and participants

All 2113 patients with T2DM attending the Diabetic Clinic of Asti, in a province in Northern Italy, were evaluated in 1996–1997, as previously reported [21, 22]. They represented 1.6% of the reference population (134 646 subjects). Since the prevalence of known T2DM was 2% in Northern Italy in that period [23], our cohort included approximately 80% of the patients with known diabetes in the study area.

All the procedures were conducted in accordance with the Declaration of Helsinki; informed consent was obtained from all the patients at baseline, and the study was approved by the Ethics Committee of the Hospital of Asti.

Baseline data of all the patients attending our clinic in 1996–1997 were obtained from the clinical records in our Diabetic Clinic from those years. At the Diabetic Clinic, all patients were examined every 4 months, the glycated hemoglobin (HbA1c) and arterial blood pressure levels were measured at each visit, and the patients received annual plasma lipid measurements and screenings for chronic complications. All laboratory measurements were centralized. Height and weight were measured by a trained nurse with the patient wearing light clothing and no shoes. The arterial blood pressure was measured by the same nurse in the morning after an overnight fast using a mercury sphygmomanometer with an appropriate cuff size, after a 5-min rest in a seated position with the arm being supported at heart level.

The values reported were the averages of the last three measurements reported in the clinical records.

Retinopathy was diagnosed via an ophthalmoscopic examination and/or retinal photography. A fundoscopy was performed through dilated pupils by an ophthalmologist who was experienced in diabetic retinopathy. In the group with retinopathy (any degree), a retinal photograph was taken, in accordance with the European protocol for diabetic retinopathy [23]. Nephropathy was established according to an albumin excretion rate of more than 20 µg/min in at least two out of three urine collections within 6 months (immunoturbidimetric method), gross proteinuria or elevated serum creatinine [24]. Distal symmetric polyneuropathy was diagnosed by the presence of neuropathic symptoms, an abnormal vibration perception threshold, the absence of ankle or knee reflexes and/or an abnormal electromyographic test. Autonomic neuropathy was diagnosed by a loss of heart rate variability or postural hypotension. A CV disease (CVD) diagnosis was based on documented events that were recorded by a physician (angina, myocardial infarction, a coronary artery bypass graft or other invasive procedures to treat coronary artery disease, transient ischemic attacks, strokes, gangrene, amputation, vascular surgery, intermittent claudication, absent foot pulses and abnormal brachial and posterior tibial blood pressures, as determined by Doppler techniques).

Information on the vital status of each patient and the causes of death of those who had died were collected from June to December 2010 from the demographic files in the town of residence or death. The underlying causes of death were determined from death certificates and coded according to the International Classification of Diseases, Ninth Revision (ICD-9) [25]. The vital status and cause of death were ascertained for 99.9% of the cohort (2112/2113). Among the causes of death, CVD (ICD 410–414, 430–438, 440), cancer (140–239), diabetes (250), and infectious diseases (001-139) were considered.

### Statistical methods

Patients were divided into tertiles according to HDL cholesterol levels and sex.

A two-sided log-rank test with an overall sample size of 2100 subjects (699 in the second HDL tertile and 1414 in the lower and upper HDL tertiles) achieves 80% power at a 0.05 significance level to detect a hazard ratio of 1.20 when the reference group hazard ratio is 1.00. ANOVA, the Kruskal–Wallis test or the Chi square test was used as appropriate to compare the clinical characteristics of the patients among tertile groups. The Kaplan–Meier curves were assessed by the log rank test and plotted by the HDL tertiles. The Cox regression model was used for survival analysis (all-cause mortality). Fine and Gray’s hazard model was used for the competing risk analyses (cause-specific mortality). The estimates were adjusted for sex, age, age at diabetes diagnosis, alcohol intake, smoking habits, values of HbA1c, non-HDL cholesterol (calculated as the difference between total cholesterol and HDL cholesterol), creatinine clearance, presence of CVDs at baseline, and treatment with sulfonylureas, insulin and hypolipemic drugs. The middle HDL cholesterol tertile was used as a reference.

## Results

The HDL cholesterol cut-off points of the tertiles were 37.5 mg/dL and 47.5 mg/dL for males and 41.5 mg/dL and 52.0 mg/dL for females, respectively (Table 1). Patients in the lower HDL cholesterol tertile were younger, more frequently insulin-treated (31.4%), and showed increased values of BMI, triglycerides, uric acid, C-peptide, prevalence of metabolic syndrome and lower total cholesterol, statin (3.0%) and metformin (69.0%) treatment rates.

**Table 1:** Characteristics of the T2DM patients by HDL cholesterol tertiles

	Lower tertile < 41.5 mg/dL F < 37.5 mg/dL M	Middle tertile 41.5 ≤ and < 52 mg/dL F 37.5 ≤ and < 47.5 mg/dL M	Higher tertile ≥52 mg/dL F ≥47.5 mg/dL M	P
Number	700	699	714	
Males (%)	45.3	45.2	43.7	0.79
Age (years)	64.6 ± 11.8	65.8 ± 10.4	67.6 ± 10.1	< 0.001
Age of DM diagnosis	55.8 ± 12.5	55.4 ± 10.7	56.1 ± 10.7	0.57
Smoking (%)	16.7	15.6	12.9	0.12
Treated with insulin (%)	31.4	23.9	27.0	0.007
Treated with sulfonylureas (%)	64.4	69.4	67.9	0.13
Treated with metformin (%)	69.0	65.8	72.3	0.032
Treated with statins (%)	3.0	6.9	9.0	<0.001
Treated with fibrates (%)	8.4	4.0	2.1	< 0.001
Treated with hypotensive drugs (%)	52.0	53.4	51.1	0.70
Treated with ACE-inhibitors	33.6	33.6	29.7	0.19
Treated with acetylsalicylic acid (%)	33.4	32.9	29.0	0.15
BMI (kg/m^2^)	29.4 ± 5.5	29.2 ± 5.1	27.6 ± 4.7	< 0.001
Systolic blood pressure (mmHg)	143.2 ± 11.4	143.6 ± 11.3	144.3 ± 11.1	0.21
Diastolic blood pressure (mmHg)	83.4 ± 4.5	83.0 ± 4.2	83.2 ± 4.5	0.23
HbA1c (%)	6.7 ± 1.5	6.6 ± 1.2	6.5 ± 1.2	0.12
C-peptide (ng/mL) *	2.3 ± 1.4	2.0 ± 1.2	1.8 ± 1.1	< 0.001
Total cholesterol (mg/dL)	198.2 ± 47.0	209.1 ± 39.5	213.4 ± 40.0	< 0.001
Triglycerides (mg/dL) *	177.6 ± 118.2	139.7 ± 75.2	106.3 ± 56.8	< 0.001
Uric acid (mg/dL)	5.5 ± 1.7	5.3 ± 1.5	5.0 ± 1.4	< 0.001
Creatinine clearance (mL/min)	107.1 ± 48.3	106.9 ± 42.6	105.8 ± 40.0	0.83
Retinopathy (%)	20.9	20.9	21.9	0.87
Nephropathy (%)	22.7	22.0	21.4	0.84
Neuropathy (%)	9.6	9.3	8.5	0.78
Coronary artery diseases (%)	21.3	20.0	16.5	0.06
Peripheral vascular diseases (%)	18.1	17.0	16.1	0.59
Metabolic syndrome (%)	64.1	45.6	33.5	< 0.001

*Kruskal–Wallis test

**Fig. 1:**
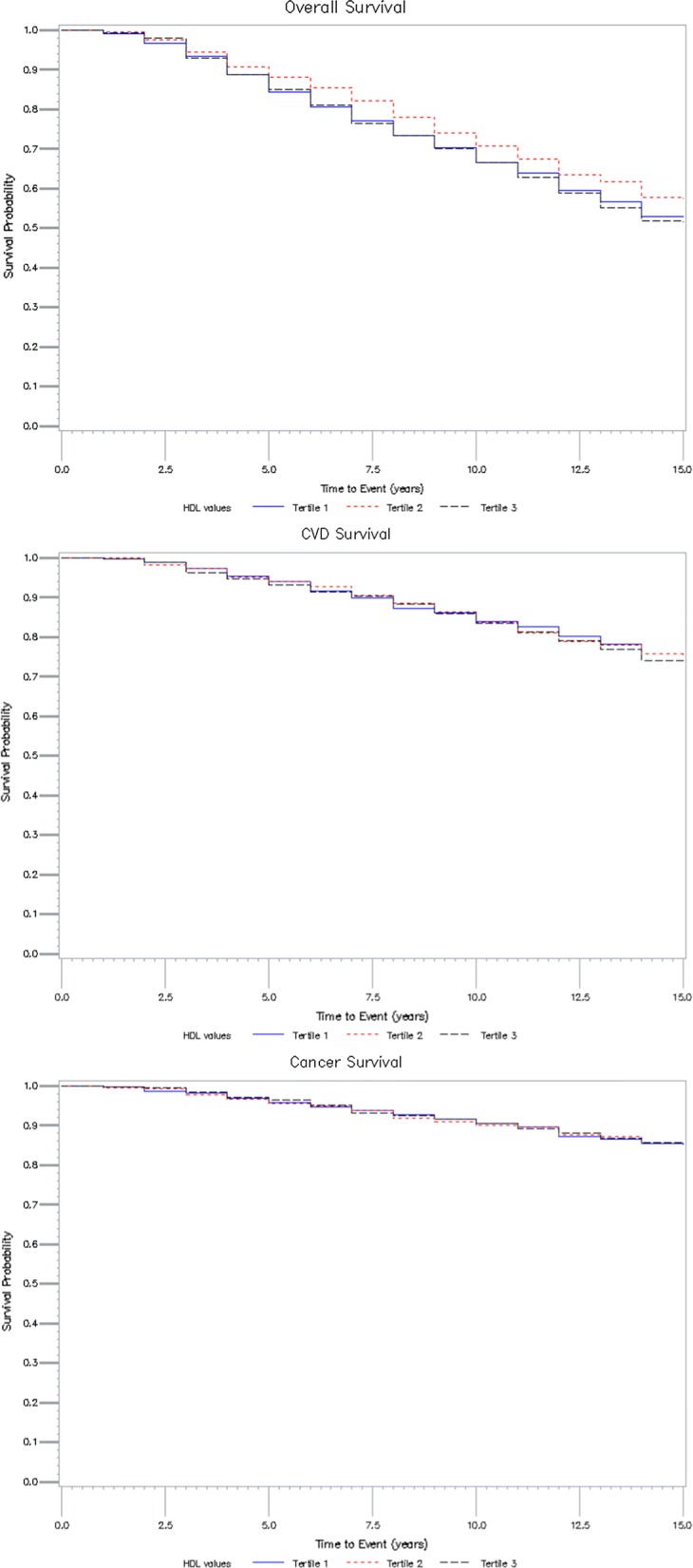
Kaplan-Meyer curves relative to overall, CVD and cancer survival by HDL tertiles. In blue, the first (lower) tertile, in dotted red, the second (middle) tertile, in dotted black, the third (higher) tertile

During a 14-year follow-up, 973 of 2112 patients died (46.1%). The survival analysis showed non-linear associations for all outcomes (Table 2). Patients belonging to the lower and to the upper HDL cholesterol tertiles did not show significant differences in all-cause, CV or cancer mortality with respect to the reference category, even if a slight increase in all-cause mortality was evident in those two tertiles. Mortality associated with infectious diseases was significantly higher in both these groups when compared to the middle tertile (Table 2), while mortality related to diabetes was significantly associated only with the upper HDL cholesterol tertile. No significant association was found between HDL cholesterol and mortality for causes other than those indicated above. The Kaplan–Meier curves for overall, CVD and cancer survival are shown in Fig.1.

**Table 2:** Adjusted hazard ratios for all cause and cause-specific mortality in type 2 diabetic patients by HDL cholesterol tertiles

Mortality	Lower tertile	p	Middle tertile	Upper tertile	p
All causes					
Number of deaths	330		297	346	
HR^a^ (95% CI)	1.12 (0.96–1.32)	0.15	1	1.11 (0.95–1.30)	0.21
Cardiovascular diseases					
Number of deaths	148		152	158	
HR^b^ (95% CI)	0.97 (0.77–1.23)	0.80	1	0.94 (0.75–1.18)	0.60
Cancer					
Number of deaths	85		87	84	
HR^b^ (95% CI)	0.92 (0.67–1.25)	0.57	1	0.89 (0.66–1.21)	0.47
Infectious diseases					
Number of deaths	37		15	33	
HR^b^ (95% CI)	2.62 (1.44–4.74)	0.002	1	2.05 (1.09–3.85)	0.03
Diabetes					
Number of deaths	34		21	43	
HR^b^ (95% CI)	1.64 (0.94–2.87)	0.08	1	1.87 (1.10–3.15)	0.02
Other causes					
Number of deaths	26		22	28	
HR^b^ (95% CI)	1.16 (0.67–2.03)	0.60	1	1.05 (0.59–1.86)	0.87

^a^Hazard ratios adjusted for gender, age, age at diabetes diagnosis, alcohol intake, smoking habits, values of non-HDL cholesterol, HbA1c and creatinine clearance, presence of CV diseases at baseline, treatment with sulfonylureas, insulin and hypolipemic drugs

^b^Fine and Gray’s hazard model for the competing risk analyses

After the exclusion of the 646 patients affected by CVD at baseline, the results of the survival analysis did not change significantly (data not shown), with the exception of a significantly higher risk for all-cause mortality (HR = 1.28; 95% CI 1.04–1.59) and diabetes mortality (HR = 2.53; 1.14–5.61) in patients in the lower HDL cholesterol tertile. Furthermore, the data did not change after excluding the 50 patients with known chronic liver diseases from the survival analyses.

## Discussion

In this cohort of T2DM patients, we found a U-shaped relationship between HDL cholesterol levels and mortality from infectious diseases and an increased diabetes-related mortality rate in individuals in the upper HDL cholesterol tertile, but no significant associations were found between HDL cholesterol levels and all-cause, CVD and cancer mortality.

### HDL cholesterol and metabolic pattern

As expected, in our T2DM cohort, higher HDL cholesterol values were significantly associated with lower triglycerides and higher total cholesterol serum levels. These data reflect the characteristic pattern of diabetic dyslipidemia [12, 26]. Furthermore, the prevalence of metabolic syndrome in the upper HDL cholesterol tertile was lower, consistent with the lower BMI. A likely lower level of insulin resistance of those individuals could be inferred as a consequence of their lower C-peptide levels.

The main function of HDL particles is reverse cholesterol transport from peripheral tissues to the liver. HDL particles show antioxidant, anti-inflammatory and anti-thrombotic properties [27–29]. Low HDL cholesterol together with higher triglyceride blood concentrations characterize so-called *atherogenic dyslipidemia* in T2DM patients. Indeed, both hyperglycemia and insulin resistance have been shown to impact HDL particles in different ways: by altering the HDL subspecies proportion in favor of the small-dense HDL_3_ with respect to the large HDL_2_; by altering the HDL proteome; by modifying the enzymatic activity of HDL-associated proteins, such as ﻿lecithin-cholesterol acyltransferase (LCAT) and paraoxonase-1 (PON-1); by increasing HDL hepatic catabolism through an enhanced activity of hepatic lipase; and by reducing their anti-inflammatory activity mediated by apolipoprotein A1 (ApoA-I) [13–17]. All these alterations contribute to hindering the anti-inflammatory and anti-atherogenic activity of HDL in T2DM patients.

### All-cause and specific-cause mortality

We would have expected lower overall and cause-specific CV mortality in patients in the upper HDL cholesterol tertile due to their overall better metabolic pattern. In contrast, all-cause and CV-related mortalities were not significantly affected by HDL cholesterol levels in our whole cohort. However, patients without CVD at baseline in the lower HDL tertile showed a slightly increased risk for all-cause mortality, thus confirming the well-known negative role of low HDL cholesterol serum concentration in individuals in primary prevention [30]. The effectiveness of HDL cholesterol quantity as a trustworthy marker for CV protection has been recently questioned [2]. Mutant ApoA-I Milano is a paradigmatic example of this incongruence: carriers of this mutation display very low levels of HDL cholesterol and are markedly protected from CVD [31]. Attempts to increase HDL cholesterol levels via cholesteryl ester transfer protein (CETP) inhibitors such as torcetrapib failed to confer atheroprotection [32, 33].

A 2-to-threefold increased risk for all-cause mortality has been reported in Danish individuals with very high HDL cholesterol levels (> 97 mg/dL for males, > 116 mg/dL for females) when compared to the group with the lowest risk (i.e. males with HDL cholesterol values of 58–76 mg/dL and females with HDL cholesterol values of 77–96 mg/dL) [7]. A slightly higher, though not statistically significant, risk for all-cause and cancer mortality was found in south Korean adults with HDL cholesterol levels > 85 mg/dL [6]. Increased all-cause (HR = 1.56; 95% CI 1.08–2.26), CV (HR = 1.62; 0.86–3.05) and non-CV deaths (HR = 1.45; 0.93–2.27) were found in US elderly individuals with HDL cholesterol levels > 90 mg/dL [9]. In an American prospective study, high HDL cholesterol levels (80–100 mg/dL) were found to be associated with an increased risk for overall (RR = 1.25; 1.09–1.49), coronary heart disease (RR = 1.09; 1.02–1.32) and stroke (RR = 1.11; 1.02–1.32) mortalities [10].

The high heterogeneity of HDL subspecies accounts for their different capabilities in reverse cholesterol transport and in atheroprotection [34, 35]. In the presence of chronic subclinical inflammation, such as in coronary artery disease, impaired HDL vessel-protecting functions have been reported even without alteration in HDL cholesterol blood concentrations, which may remain in the normal range [2, 4, 5, 7]. Similarly, in T2DM patients, characterized by chronic subclinical inflammation status and by the aforementioned atherogenic dyslipidemia, reaching normal or even high HDL cholesterol levels might not be effective in conferring atheroprotection [4, 13]. Only one study specifically addressed the relationships between HDL cholesterol levels and major outcomes in T2DM patients, showing a direct association between HDL cholesterol and all-cause mortality (HR = 1.14; 1.07–1.22 per 4 mg/dL HDL cholesterol increase), CV mortality (HR = 1.12; 1.03–1.22) and CV fatal and nonfatal events (HR = 1.10; 1.02–1.18) in the presence of LDL cholesterol levels < 77 mg/dL. In contrast, in patients with LDL cholesterol levels ranging between 77 and 97 mg/dL, higher HDL cholesterol was associated with a lower CV risk (HR = 0.85; 0.75–0.95 per 4 mg/dL HDL cholesterol increase) [8]. These counterintuitive relationships have been explained by the authors as possible confounding effects of statin treatment, which is responsible for both the reduction in LDL cholesterol serum concentration and the inhibition of hepatic lipase, a key enzyme in HDL catabolism. A reduction in lipase activity is indeed associated with an increase in HDL cholesterol levels and contemporarily with an increased risk for CV events.

As previously reported, HDL function is impaired in T2DM patients, independent of HDL cholesterol serum levels [15, 16]. In our cohort, patients in the upper HDL cholesterol tertile were older than those in the other tertiles. This could have strongly impacted the evaluated outcomes. However, our results did not change after adjustment for age. If these results are confirmed by larger prospective studies, it could be hypothesized that the functional capacity (that is, the quality) of HDL particles is more important from a prognostic point of view than circulating cholesterol concentrations (that is, the quantity), at least in T2DM patients. As a consequence, criteria and targets for lipid-lowering therapies might also be reconsidered.

### Deaths from infectious diseases

We found a U-shaped association between HDL cholesterol level and mortality with infectious causes, since both lower and higher HDL cholesterol concentrations were associated with an increased risk. Moreover, a significant association was found between the higher HDL cholesterol tertile and mortality related to diabetes. Since the great majority of deaths as attributed to diabetes were associated with diabetic foot complications (data not shown), we can relate this latter finding to the infectious diseases group. Even if our results do not necessarily imply causality and are difficult to completely understand, data supporting these associations exist in the literature [36–42]. Statistically significant U-shaped associations between HDL cholesterol levels and CRP serum concentrations have been reported, even if no data about the origin of inflammation are available [10]. Other clinical studies have found relationships between HDL cholesterol values and infections, either reporting associations between low HDL cholesterol at hospital admission and an increased risk of short-term infectious complications in relation to hospitalization, identifying a linear inverse correlation between HDL cholesterol and risk of infection or sepsis [36, 37] or reporting low HDL cholesterol as a risk factor for nosocomial infections [38, 39]. To the best of our knowledge, only two population-based cohort studies have evaluated the relationships between HDL cholesterol serum concentrations and the risk of community-acquired infectious diseases or sepsis. One study failed to identify an association between HDL cholesterol and the risk for long-term sepsis [40]. The second study, carried out with more than 100,000 individuals, found a U-shaped relationship between HDL cholesterol levels and infectious diseases requiring hospitalization and deaths due to infections; in particular bacterial infections (pneumonia, cutaneous and urinary infections) were associated with both lower and higher HDL cholesterol, and viral infections were associated only with lower HDL cholesterol values [41].

Both low (HDL cholesterol < 31 mg/dL) and high (HDL cholesterol > 100 mg/dL) concentrations of HDL cholesterol significantly increased the risk for infectious disease-related admissions and mortality.

HDL particles are known to confer protection against bacterial and parasitic infections via several biochemical pathways. Preclinical studies have demonstrated that lipopolysaccharides from gram-negative bacteria and lipoteichoic acid from gram-positive bacteria are bound and cleared by HDL particles via ApoA-I [43–45]. Therefore, the inability to remove bacterial toxins from the bloodstream might explain the increased risk for infectious diseases and related adverse events in individuals with low HDL cholesterol values. Moreover, HDL particles regulate innate and adaptive immunity at different stages, from controlling hematopoiesis to modulating circulating leucocyte activity [46]. The acute phase reaction induced by infections promptly induces major structural and functional changes in HDL particles that lead to a loss of anti-inflammatory properties [3, 47–49]. Hence, dysfunctional HDL particles could fail to sustain the immune system to prevent the unfavorable consequences of sepsis.

On the other hand, very little is known about the mechanisms that might underlie the unfavorable effects of high HDL cholesterol levels on the risk of infectious diseases. Very high HDL cholesterol levels could be due to genetic polymorphisms that are related both to higher HDL cholesterol concentrations and to increased disease susceptibility, although no direct causality can be inferred [50, 51]. During the process of infection, patients displaying high HDL cholesterol levels might undergo an important displacement of constitutive ApoA-I, the major anti-inflammatory element of HDL particles, mediated by the acute-phase proteins serum amyloid A (SAA) and secretory phospholipase A2 (sPLA2), in a way that could amplify systemic inflammation [52].

### Limitations of the study

This study suffers from some limitations. Neither data about inflammatory status, which could be helpful in better understanding its relationship with the lipidic profile, nor data related to the specific infectious diseases were available. Markers of HDL functionality, HDL subspecies and apolipoproteins, and cholesterol efflux capacity were not studied. Only baseline lipid values were collected. Indeed, misclassification of the patients could have reduced the association found.

The possibility of residual confounding factors could not be excluded owing to the observational nature of the study. The long follow-up period could have influenced the results. Finally, comparing studies with different cut-off values and reference ranges limits the interpretation of the existing literature.

The strengths of our study are the representativeness of our cohort of T2DM patients in the study area and the length and completeness of the follow-up.

## Conclusion

We found a significant U-shaped association between HDL cholesterol and mortality associated with infectious diseases in a population-based cohort of T2DM patients, but no statistically significant associations were found between HDL cholesterol and all-cause and CV and cancer mortality. These results, which need to be confirmed by further studies, corroborate the hypothesis that HDL cholesterol levels are nonprotective in T2DM patients.

## Data Availability

The datasets used and/or analyzed during the current study are available from the corresponding author on reasonable request.

## Abbreviations

ApoA-I: Apolipoprotein-A1
CETP: Cholesteryl esters transfer protein
CV: Cardiovascular
CVD: Cardiovascular disease
HR: Hazard ratio
LCAT: Lecithin-cholesterol acyltransferase
PON-1: Paraoxonase-1
SAA: Serum amyloid A
sPLA2: Secretory phospholipase A2
T2DM: Type 2 diabetes mellitus

## References

[1] Santos RD, Barter PJ (2019). HDL cholesterol level and mortality occurrence in the elderly: is the good cholesterol always good?. J Clin Endocrinol Metab.

[2] Nessler K, Windak A, Grzybczak R, Nessler MB, Siniarski A, Gajos G (2018). High-density lipoprotein (Hdl) cholesterol—more complicated than we think?. Ann Agric Environ Med.

[3] Tall AR, Yvan-Charvet L (2015). Cholesterol, inflammation and innate immunity. Nat Rev Immunol.

[4] Ragbir S, Farmer JA (2010). Dysfunctional high-density lipoprotein and atherosclerosis. Curr Atherosc Rep..

[5] Pérez-Méndez Ó, Pacheco HG, Martínez-Sánchez C, Franco M (2014). HDL-cholesterol in coronary artery disease risk: function or structure?. Clin Chim Acta.

[6] Oh IH, Hur JK, Ryoo JH, Jung JY, Park SK, Yang HJ (2019). Very high high-density lipoprotein cholesterol is associated with increased all-cause mortality in South Koreans. Atherosclerosis..

[7] Madsen CM, Varbo A, Nordestgaard BG (2017). Extreme high high-density lipoprotein cholesterol is paradoxically associated with high mortality in men and women: two prospective cohort studies. Eur Heart J.

[8] Sharif S, Van Der Graaf Y, Nathoe HM, De Valk HW, Visseren FLJ, Westerink J (2016). HDL cholesterol as a residual risk factor for vascular events and all cause mortality in patients with type 2 diabetes. Diabetes Care.

[9] Li ZH, Lv Y, Zhong WF, Gao X, Kraus V, Zou MC (2019). High-density lipoprotein cholesterol and all-cause and cause-specific mortality among the elderly. J Clin Endocrinol Metab.

[10] Mazidi M, Mikhailidis DP, Banach M (2019). Associations between risk of overall mortality, cause-specific mortality and level of inflammatory factors with extremely low and high high-density lipoprotein cholesterol levels among American adults. Int J Cardiol.

[11] Bowe B, Xie Y, Xian H, Balasubramanian S, Zayed MA, Al-Aly Z (2016). High density lipoprotein cholesterol and the risk of all-cause mortality among U.S. veterans. Clin J Am Soc Nephrol.

[12] Hermans MP, Valensi P (2018). Elevated triglycerides and low high-density lipoprotein cholesterol level as marker of very high risk in type 2 diabetes. Curr Opin Endocrinol Diabetes Obes.

[13] Vergès B (2015). Pathophysiology of diabetic dyslipidaemia: where are we?. Diabetologia.

[14] Davidson WS, Shah AS (2019). High-density lipoprotein subspecies in health and human disease: focus on type 2 diabetes. Methodist Debakey Cardiovasc J..

[15] Lemmers RFH, van Hoek M, Lieverse AG, Verhoeven AJM, Sijbrands EJG, Mulder MT (2017). The anti-inflammatory function of high-density lipoprotein in type II diabetes: a systematic review. J Clin Lipidol..

[16] Femlak M, Gluba-Brzózka A, Ciałkowska-Rysz A, Rysz J (2017). The role and function of HDL in patients with diabetes mellitus and the related cardiovascular risk. Lipids Health Dis..

[17] Zhang P, Gao J, Pu C, Zhang Y (2017). Apolipoprotein status in type 2 diabetes mellitus and its complications (Review). Mol Med Rep..

[18] Scott R, O’Brien R, Fulcher G, Pardy C, D’Emden M, Tse D (2009). Effects of fenofibrate treatment on cardiovascular disease risk in 9,795 individuals with type 2 diabetes and various components of the metabolic syndrome the fenofibrate intervention and event lowering in diabetes (FIELD) study. Diabetes Care.

[19] Annuzzi G, Giacco R, Patti L, Di Marino L, De Natale C, Costabile G (2008). Postprandial chylomicrons and adipose tissue lipoprotein lipase are altered in type 2 diabetes independently of obesity and whole-body insulin resistance. Nutr Metab Cardiovasc Dis..

[20] Mach F, Baigent C, Catapano AL, Koskina KC, Casula M, Badimon L (2019). 2019 ESC/EAS guidelines for the management of dyslipidaemias: lipid modification to reduce cardiovascular risk. Atherosclerosis..

[21] Bo S, Cavallo-Perin P, Gentile L, Repetti E, Pagano G (2000). Relationship of residual beta-cell function, metabolic control and chronic complications in type 2 diabetes mellitus. Acta Diabetol.

[22] Bo S, Cavallo-Perin P, Gentile L (1999). Prevalence of patients reaching the targets of good control in normal clinical practice: a cohort-based study in type 2 diabetes [9]. Diabetes Care.

[23] Bo S, Gentile L, Castiglione A, Prandi V, Canil S, Ghigo E (2012). C-peptide and the risk for incident complications and mortality in type 2 diabetic patients: a retrospective cohort study after a 14-year follow-up. Eur J Endocrinol.

[24] Persson F, Rossing P (2018). Diagnosis of diabetic kidney disease: state of the art and future perspective. Kidney Int Suppl..

[25] International Classification of Diseases, Ninth Revision (ICD-9). ftp://ftp.cdc.gov/pub/Health_Statistics/NCHS/Publications/ICD-9. Accessed 3 Mar 2020. 10.7326/0003-4819-88-3-424 629506

[26] Wu L, Parhofer KG (2014). Diabetic dyslipidemia. Metabolism.

[27] Säemann MD, Poglitsch M, Kopecky C, Haidinger M, Hörl WH, Weichhart T (2010). The versatility of HDL: a crucial anti-inflammatory regulator. Eur J Clin Invest.

[28] Tsompanidi EM, Brinkmeier MS, Fotiadou EH, Giakoumi SM, Kypreos KE (2010). HDL biogenesis and functions: role of HDL quality and quantity in atherosclerosis. Atherosclerosis..

[29] Santos-Gallego CG, Rosenson RS (2014). Role of HDL in Those with Diabetes. Curr Cardiol Rep.

[30] Vitali C, Khetarpal SA, Rader DJ (2017). HDL cholesterol metabolism and the risk of CHD: new insights from human genetics. Curr Cardiol Rep.

[31] Roma P, Gregg RE, Meng MS, Ronan R, Zech LA, Franceschini G (1993). In vivo metabolism of a mutant form of apolipoprotein A-I, apo A- I(Milano), associated with familial hypoalphalipoproteinemia. J Clin Invest..

[32] Barter PJ, Caulfield M, Eriksson M, Grundy SM, Kastelein JJP, Komajda M (2007). Effects of torcetrapib in patients at high risk for coronary events. N Engl J Med.

[33] Kastelein JJP, Van Leuven SI, Burgess L, Evans GW, Kuivenhoven JA, Barter PJ (2007). Effect of torcetrapib on carotid atherosclerosis in familial hypercholesterolemia. N Engl J Med.

[34] Davidson WS, Silva RAGD, Chantepie S, Lagor WR, Chapman MJ, Kontush A (2009). Proteomic analysis of defined hdl subpopulations reveals particle-specific protein clusters: relevance to antioxidative function. Arterioscler Thromb Vasc Biol.

[35] Gordon SM, Deng J, Lu LJ, Davidson WS (2010). Proteomic characterization of human plasma high density lipoprotein fractionated by gel filtration chromatography. J Proteome Res.

[36] Grion CMC, Cardoso LTQ, Perazolo TF, Garcia AS, Barbosa DS, Morimoto HK (2010). Lipoproteins and CETP levels as risk factors for severe sepsis in hospitalized patients. Eur J Clin Invest.

[37] Shor R, Wainstein J, Oz D, Boaz M, Matas Z, Fux A (2008). Low HDL levels and the risk of death, sepsis and malignancy. Clin Res Cardiol.

[38] Canturk NZ, Canturk Z, Okay E, Yirmibesoglu O, Eraldemir B (2002). Risk of nosocomial infections and effects of total cholesterol, HDL cholesterol in surgical patients. Clin Nutr.

[39] Rodríguez-Sanz A, Fuentes B, Martínez-Sánchez P, Prefasi D, Martínez-Martínez M, Correas E (2013). High-density lipoprotein: a novel marker for risk of in-hospital infection in acute ischemic stroke patients?. Cerebrovasc Dis..

[40] Guirgis FW, Donnelly JP, Dodani S, Howard G, Safford MM, Levitan EB (2016). Cholesterol levels and long-term rates of community-acquired sepsis. Crit Care.

[41] Madsen CM, Varbo A, Tybjærg-Hansen A, Frikke-Schmidt R, Nordestgaard BG, Tybjaerg-Hansen A (2018). U-shaped relationship of HDL and risk of infectious disease: two prospective population-based cohort studies. Eur Heart J.

[42] Carey IM, Critchley JA, Dewilde S, Harris T, Hosking FJ, Cook DG (2018). Risk of infection in type 1 and type 2 diabetes compared with the general population: a matched cohort study. Diabetes Care.

[43] Wurfel MM, Kunitake ST, Lichenstein H, Kane JP, Wright SD (1994). Lipopolysaccharide (LPS)-binding protein is carried on lipoproteins and acts as a cofactor in the neutralization of LPS. J Exp Med.

[44] Levels JHM, Abraham PR, Van Barreveld EP, Meijers JCM, Van Deventer SJH (2003). Distribution and kinetics of lipoprotein-bound lipoteichoic acid. Infect Immun.

[45] Guo L, Ai J, Zheng Z, Howatt DA, Daugherty A, Huang B (2013). High density lipoprotein protects against polymicrobe-induced sepsis in mice. J Biol Chem.

[46] Catapano AL, Pirillo A, Bonacina F, Norata GD (2014). HDL in innate and adaptive immunity. Cardiovasc Res.

[47] Kumon Y, Nakauchi Y, Kidawara K, Fukushima M, Kobayashi S, Ikeda Y (1998). A longitudinal analysis of alteration in lecithin-cholesterol acyltransferase and paraoxonase activities following laparoscopic cholecystectomy relative to other parameters of HDL function and the acute phase response. Scand J Immunol.

[48] Feingold KR, Grunfeld C (2010). The acute phase response inhibits reverse cholesterol transport. J Lipid Res.

[49] Pirillo A, Catapano AL, Norata GD, von Eckardstein A, Kardassis D (2015). HDL in infectious diseases and sepsis. High density lipoproteins: from biological understanding to clinical exploitation.

[50] Zanoni P, Khetarpal SA, Larach DB, Hancock-Cerutti WF, Millar JS, Cuchel M (2016). Rare variant in scavenger receptor BI raises HDL cholesterol and increases risk of coronary heart disease. Science.

[51] Agerholm-Larsen B, Nordestgaard BG, Steffensen R, Jensen G, Tybjærg-Hansen A (2000). Elevated HDL cholesterol is a risk factor for ischemic heart disease in white women when caused by a common mutation in the cholesteryl ester transfer protein gene. Circulation.

[52] Jahangiri A, De Beer MC, Noffsinger V, Tannock LR, Ramaiah C, Webb NR (2009). HDL remodeling during the acute phase response. Arterioscler Thromb Vasc Biol.

